# *PA2146* Gene Knockout Is Associated With *Pseudomonas aeruginosa* Pathogenicity in Macrophage and Host Immune Response

**DOI:** 10.3389/fcimb.2020.559803

**Published:** 2020-10-07

**Authors:** Pengfei She, Yiqing Liu, Zhen Luo, Lihua Chen, Linying Zhou, Zubair Hussain, Yong Wu

**Affiliations:** Department of Clinical Laboratory, The Third Xiangya Hospital of Central South University, Changsha, China

**Keywords:** *PA2146*, virulence factor, *Pseudomonas aeruginosa*, macrophage, immune response, regulation

## Abstract

*Pseudomonas aeruginosa* is a common gram-negative bacterium that usually causes nosocomial infection. The main pathogenicity of *P. aeruginosa* is caused by its virulence factors. *PA2146* is reported to be a potential virulence-regulating gene and is highly expressed in the biofilms of *P. aeruginosa*. However, the effect of *PA2146* mutant (PAO1Δ*PA2146*) on the macrophage immune response and murine models has not been reported. In the present study, *PA2146* knockout was performed by homologous recombination. We found that PAO1Δ*PA2146* stimulation significantly increased pyocyanin production but inhibited interleukin-6 secretion by neutrophils compared to PAO1 stimulation. In addition, PAO1Δ*PA2146* treatment significantly inhibited cytokine production in macrophages independent of cell killing. In an acute pneumonia murine infection model, treatment with *P. aeruginosa* infected with PAO1Δ*PA2146* inhibited cytokine secretion in the lungs but increased the infiltration of inflammatory cells compared to the wild-type group. The paradoxical results indicate that *PA2146* deletion may also increase the production of virulence factors other than pyocyanin, which may not only increase inflammatory cell infiltration in the lungs but also lead to immune cells “shock.” Overall, our findings suggest that *PA2146* could serve as a *P. aeruginosa* virulence-regulating gene that regulates its macrophage and host immune response.

## Introduction

*Pseudomonas aeruginosa* is a conditional pathogen that causes various infections in immunosuppressed patients (Mulcahy et al., 2014). As of 2015, *P. aeruginosa, Escherichia coli* (*E. coli*), and *Klebsiella pneumoniae* accounted for 70% of Gram-negative hospital infections and maintained their status of “serious threats” in 2019 (Svečnjak et al., 2020). *P. aeruginosa* can form biofilms on the surfaces of surgical instruments and human tissues, causing urinary tract infections, bone/joint infections, endocarditis, chronic wound infections, and cystic fibrosis, which are the major sources of morbidity and mortality in adults (Rineh et al., 2020).

The main pathogenicity of *P. aeruginosa* may be due to the production of its virulence factors (including endotoxin and exotoxin) and subsequent pathological immune response. Exotoxin pyocyanin is one of the major virulence factors secreted by *P. aeruginosa*. Pyocyanin is regulated by the quorum sensing (QS) system, an important communication system that controls survival, virulence and biofilm formation in bacterial communities (Seleem et al., 2020). The QS system is mainly regulated by four QS network sub-systems, including the *las, rhl*, PQS, and IQS systems. The hierarchical QS network plays a key role in the regulation of virulence gene expression (Wang et al., 2020).

Pyocyanin is an important QS-related virulence factor of *P. aeruginosa*, which can activate immune evasion by inhibiting the secretion of nitric oxide, tumor necrosis factor-α, and interleukin (IL)-1β in macrophages (Marreiro de Sales-Neto et al., 2019). Pyocyanin also produces superoxide anions by non-enzymatic transfer of electrons from NADH and NADPH to oxygen, which may inhibit NLRP3 inflammasome formation and cytokine secretion (Virreira Winter and Zychlinsky, 2018). Moreover, pyocyanin can directly damage respiratory epithelium cells, leading to chronic infection in patients with cystic fibrosis (CF).

Using the whole transcriptome analysis and isogeneic knockout mutants by Attila et al. (2008), PA2146 was first identified as the virulence gene in *P. aeruginosa* that may cause poplar tree infection in rhizosphere. In their study, isogenic mutation of *PA2146* cause significantly inhibit the biofilm formation ability and less virulence on poplar seed germination, but compared with the wild-type strain, it could increase the existence percentage and motility (50–83% more) on poplar tree roots. Subsequently, by using RNA sequencing technology to evaluate the gene expression difference between mature *P. aeruginosa* biofilms and planktonic cells, Dötsch et al. (2012) demonstrated that 227 genes were significantly upregulated in the 24 h biofilm compared with its plankton. Among them, *PA2146* was predominantly more highly expressed in *P. aeruginosa* PA14 biofilms, which was statistically significant compared with planktonic cells. The raw data shows that the expression level of PA2146 in 24-h biofilm was 36.75 times higher than that in planktonic cells, while it was 139.10 times higher in 48-h biofilm. However, to the best of our knowledge, there is no study reporting its biological function in immune cells and *in vivo* animal models. In the present study, *P. aeruginosa* immune evasion and *in vivo* pathogenicity were investigated in macrophages and acute pneumonia animal models, respectively.

## Materials and Methods

### Bacterial Strains, Plasmids, and Culture Conditions

The bacterial strains and plasmids used in the present study are listed in Table 1. Bacteria were stored at −80°C in 50% glycerol and recovered on sheep blood agar (BKMAM, Changsha, China) at 37°C overnight. Unless otherwise stated, *E. coli* and *P. aeruginosa* strains were cultured in Luria-Bertani (LB) medium (Solarbio, Beijing, China) at 37°C with shaking at 200 rpm.

**Table 1:** Bacterial strains and plasmids.

**Strains and plasmids**	**Description**	**Source**
**STRAINS**		
***P. aeruginosa***		
PAO1	Wild type, ATCC strain	Shan et al., 2004
PAO1Δ*PA2146*	Isogenic *PA2146* deletion mutant in PAO1	This study
***E. coli***		
DH5α	Clone host strain	Laboratory stock
β2163	Clone host strain	Laboratory stock
**PLASMIDS**		
pLP12	Shuttle cloning vector, temp sensitive (TC^r^)^a^	Laboratory stock
pLP*PA2146*	pLP12 containing fragments 636-bp upstream and 608-bp downstream of PA2146 gene, for PA2146 mutagenesis (TC^r^)	This study

a *TC^r^, tetracycline resistance*.

### Construction of *P. aeruginosa PA2146* Mutant Strain by Allelic Replacement

*PA2146* mutant strain construction was performed according to a previously described method with minor modifications (Luo et al., 2015). The primers used are listed in Table 2. Briefly, the upstream and downstream fragments of *PA2146* were amplified with *PA2146*-up-F/R and *PA2146*-down-F/R, respectively. The amplified products were then fused by overlapping PCR, and the fused fragment was cloned into pLP12 (KnoGen Biotech, Guangzhou, China) using recombinant enzyme Exnase II (ClonExpress II, Vazyme) to generate recombinant plasmid pLP*PA2146*. The resulting plasmid was then transformed into *E. coli* DH5α, and the recombinant plasmids were confirmed by PCR using the primer pair pLP-U-F/R. Next, pLP*PA2146* was transferred into *E. coli* β2163 and selected on LB agar containing 0.3 mM daptomycin (DAP) with 0.3% D-glucose. *E. coli* β2163 was then co-cultured with PAO1, and the insertion mutation was selected on LB agar containing 36 μg/mL tetracycline (TC) with 0.3% D-glucose and confirmed using the primer pair *PA2146*-up-F and *PA2146*-down-R. The PAO1Δ*PA2146* strain was selected on LB agar with 0.4% L-arabinose. The *PA2146* mutant was confirmed by sequencing using the primers *PA2146*-TF and *PA2146*-down-R.

**Table 2:** Primers used in this study.

**Primers**	**Oligonucleotide(5′ → 3′)**
**PAO1Δ*****PA2146*** **Construction**	
*PA2146*-up-F	GGAATCTAGACCTTGAGTCGGAGGTCCACGTTGCAGTCCA
*PA2146*-up-R	GGTTATCAGTTCCCGCCGTGCTGTGCCATTTCTATTTCCTCCG
*PA2146*-down-F	CGGAGGAAATAGAAATGGCACAGCACGGCGGGAACTGATAACC
*PA2146*-down-R	ACAGCTAGCGACGATATGTCCTGGATGAAGAACACCGGCA
*PA2146*-TF	AGCAGGCTGTAGTGGCTTTTCTC
pLP-U-F	GACACAGTTGTAACTGGTCCA
pLP-U-R	CAGGAACACTTAACGGCTGAC
**Primers for qPCR**	
*PA2146*-F	ATGGCACAGCATCAAGGTGG
*PA2146*-R	CGCTGCGGATCGTTCTTGA
*16srRNA*-F	TGAGATGTTGGGTTAAGTCCCGCA
*16srRNA*-R	CGGTTTCGCTGCCCTTTGTATTGT
*IL-1β*-F	TCGCAGCAGCACATCAACAAGAG
*IL-1β*-R	AGGTCCACGGGAAAGACACAGG
*IL-6*-F	CTCCCAACAGACCTGTCTATAC
*IL-6*-R	CCATTGCACAACTCTTTTCTCA
*MIP-2*-F	GGTTGACTTCAAGAACATCCAG
*MIP-2*-R	TTGAGAGTGGCTATGACTTCTG
*GAPDH*-F	AACTTTGGCATTGTGGAAGG
*GAPDH*-R	CAGGGTCAAGGCAAGCCTC

### Detection of Pyocyanin Pigment

Experiment was performed based on our previous report (Qu et al., 2016). Briefly, the fresh colonies of *P. aeruginosa* were sub-cultured in LB broth, incubated at 37°C at 180 rpm for 16 h, centrifuged at 4,000 × g for 15 min to collect the supernatant, and used a 0.22 μm filter membrane Sterilize (Millipore, USA). The pyocyanin pigment was extracted with chloroform (in the ratio of 2: 3), and then reextracted with 1 mL of 0.2 mol/L hydrogen chloride. The absorbance at 540 nm (A540) was read as the relative quantity of pyocyanin pigment.

### RAW264.7 Intracellular Killing Assay

Murine macrophage-like RAW264.7 cells (Xiangya Hospital, Changsha, China) were cultured in Dulbecco's modified Eagle's medium with 10% fetal bovine serum (Gibco, USA) and 1% L-glutamine at 37°C in 5% CO_2_. RAW264.7 cells were seeded into six-well plates (Corning/Costar, USA) at a concentration of 1 × 10^5^ cells/well. After 24 h of incubation, the cells were infected with *P. aeruginosa* at a multiplicity of infection (MOI) of 10, and further cultured for 2–4 h. The cells and supernatant were mixed with tips and 1 ml of sterile saline containing 1% Triton X (Solarbio, Beijing, China) was added to the suspension, the total number of bacteria was determined by counting colony forming units (CFUs) on sheep blood agar at the time point of 2, 3, and 4 h. As for intracellular bacterial counting, cells were washed with phosphate buffered saline (PBS) and added media with 100 μg/ml gentamicin (Solarbio, Beijing, China) to eradicate any extracellular bacteria. Cells were washed with PBS and lysed in 1 ml of sterile saline containing 0.5% Triton X. The number of intracellular bacterial load in the lysates was also determined by counting CFUs (Fu et al., 2017). As for intraphagocytic bacteria observation, cells were washed with PBS and stained with 2% safranin solution (BASO, Zhuhai, China) and observed with a microscopy.

### RAW264.7 Cell Viability Detection by CCK-8 Kit

Overnight cultures of *P. aeruginosa* PAO1 and PAO1Δ*PA2146* were diluted with fresh LB broth and sub-cultured at 37°C for a log-phase growth period at 200 rpm. The culture supernatant was collected by centrifugation at 4,000 × g for 15 min and sterilized with a 0.22 μm filter. RAW264.7 cells were seeded into six-well plates at a concentration of 1 × 10^5^ cells/well. After 24 h of incubation, the culture medium was replaced with the bacterial supernatant and further cultured for 3 h. The cells treated with LB broth were set as a control group. Then, the cell viability of RAW264.7 was detected by a CCK-8 kit (Dojindo, Japan) strictly following the manufacturer's instructions.

### RNA Extraction and Quantitative Reverse Transcription-PCR (qRT-PCR)

The expression of *PA2146* was determined *in P. aeruginosa*. Overnight cultures of *P. aeruginosa* were diluted with fresh LB broth and sub-cultured at 37°C at 200 rpm for a log-phase growth period. After that, the suspension was adjusted to ~1 ×10^6^ CFU/mL with LB broth, and 2 mL of the culture was transferred to a 6-well plate. After incubation at 37°C for 8 and 16 h, bacteria in the supernatant were collected as planktonic cells in EP tubes by centrifugation at 12,000 rpm for 5 min, and then the plate was washed twice with PBS to remove planktonic cells. The biofilms that adhered to the wells were collected by scraping and centrifuging at 12,000 rpm for 5 min. RNA extraction was performed with the E.Z.N.A. Total RNA Kit II (Omega Bio-tek, Norcross, GA). One microliter of RNA was used for cDNA synthesis with TransScript All-in-One First-Strand cDNA Synthesis SuperMix for qPCR (Transgene, Beijing, China). Quantitative PCR was performed using TransStart Green qPCR SuperMix UDG (Transgene, Beijing, China) on a real-time quantitative PCR system (Eppendorf, Germany).

Next, cytokine expression was evaluated in RAW264.7 cells. Cells were treated as described above in a 6-well plate, collected with a scraper, and washed twice with PBS. *TransZol*^TM^ Up (Transgene, Beijing, China) was used for cell lysis, and 200 μL chloroform was added to remove organic soluble substances. The RNA was precipitated with isopropanol (500 μ L). After centrifugation at 10,000 g for 10 min at 4°C, the precipitated RNA was washed with 75% ethanol and dissolved in DEPC water. Then cDNA synthesis and quantitative PCR were performed as described above. All primers used in qRT-PCR are designed and synthesized by Sangon Biotech. (Shanghai, China) and are listed in Table 2.

### Cytokines Determination by ELISA

PAO1 and its mutant strain PAO1Δ*PA2146* were cultured in LB broth at 37°C overnight. The supernatant was collected by centrifugation at 4,000 g for 15 min and filtered with a 0.22 μm filter. RAW264.7 and THP-1 cells were seeded into 96-well plates (Corning/Costar, USA) at a concentration of 1 × 10^5^ cells/well with 100% supernatants of PAO1 and PAO1Δ*PA2146*, and the cells in LB broth were used as the control. After incubation in 5% CO_2_ at 37°C for 3 h, the secretion of cytokines (IL-1β, IL-6, and MIP-2) in RAW264.7 and THP-1 supernatants were detected using ELISA kits (all from Abcam Inc. USA) as per the manufacturer's instructions. The detection limits were 1 pg/mL for IL-1β, 2 pg/mL for IL-6, and 3.13 pg/mL for MIP-2 (Zhu et al., 2013).

### Bacterial Growth Curve

Overnight cultures of *P. aeruginosa* (PAO1 and PAO1Δ*PA2146*) were diluted with LB broth to ~1 × 10^5^ CFU/mL and cultured in a 50 mL centrifuge tube at 37°C at 180 rpm. The turbidity of the culture medium was detected with a microplate reader (Bio-Rad, USA) at 630 nm at intervals of 2 or 4 h for a total of 72 h.

### Mouse Model of Acute Pneumonia

All animal studies were approved by the Institutional Animal Care and Use Committee of Central South University, and animal care and experiments were performed in accordance with the guidelines and regulations approved by the Administration of Affairs Concerning Experimental Animals in China. The acute pneumonia model was established as described previously (Smith et al., 2018). Female 6 to 7-week-old BALB/c mice were purchased from Hunan Slake Jingda Experimental Animal Co., Ltd. (Changsha, China). Mice were anesthetized by intraperitoneal injection of sodium pentobarbital (50 mg/kg), and infected with *P. aeruginosa* PAO1 and PAO1Δ*PA2146* via trachea perfusion at a concentration of 5 × 10^6^ CFU/50 μL. In the vehicle group, mice were treated with 50 μL PBS. The surviving mice were recorded at 6 h intervals for a total of 144 h (~6 days).

For pathological analysis, the mice were sacrificed 20 h after infection, and the lungs were removed and photographed with a camera (NIKON, Japan). For histologic analysis, the mice lungs were fixed in 4% buffered formaldehyde for 24 h and embedded in paraffin. Then the lung tissue sections (3 μm thick) were prepared, stained with haematoxylin and eosin (H&E), and visualized under a NIKON ECLIPSE Ci microscope via the Digital Sight DS-FI2 monitor (Japan). Inflammation score was defined as: 0, no inflammation; 1, mild inflammation: inflammation area <20% of the total lung tissue area; 2, moderate inflammation: 20–50% of the total lung tissue area; 3, severe inflammation: inflammation area accounts for more than 50% of the total lung tissue area. Next, the lung tissues were homogenized and analyzed for the secretion of cytokines (IL-1β, IL-6, and MIP-2) using ELISA kits (all from Abcam Inc. USA) and plated in serial dilutions for obtaining CFU counts.

### Statistical Analysis

Statistical analysis was performed using GraphPad Prism 8.0 (GraphPad Software, CA, USA). Analysis of variance (ANOVA) was used to compare two different groups, and Tukey's multiple comparison test was used to compare differences among three groups. All experiments were performed in biological triplicates. *P* < 0.05 indicated statistical significance.

## Results

### *PA2146* Knockout Promotes *P. aeruginosa* Virulence Factor Production

**Figure 1:**
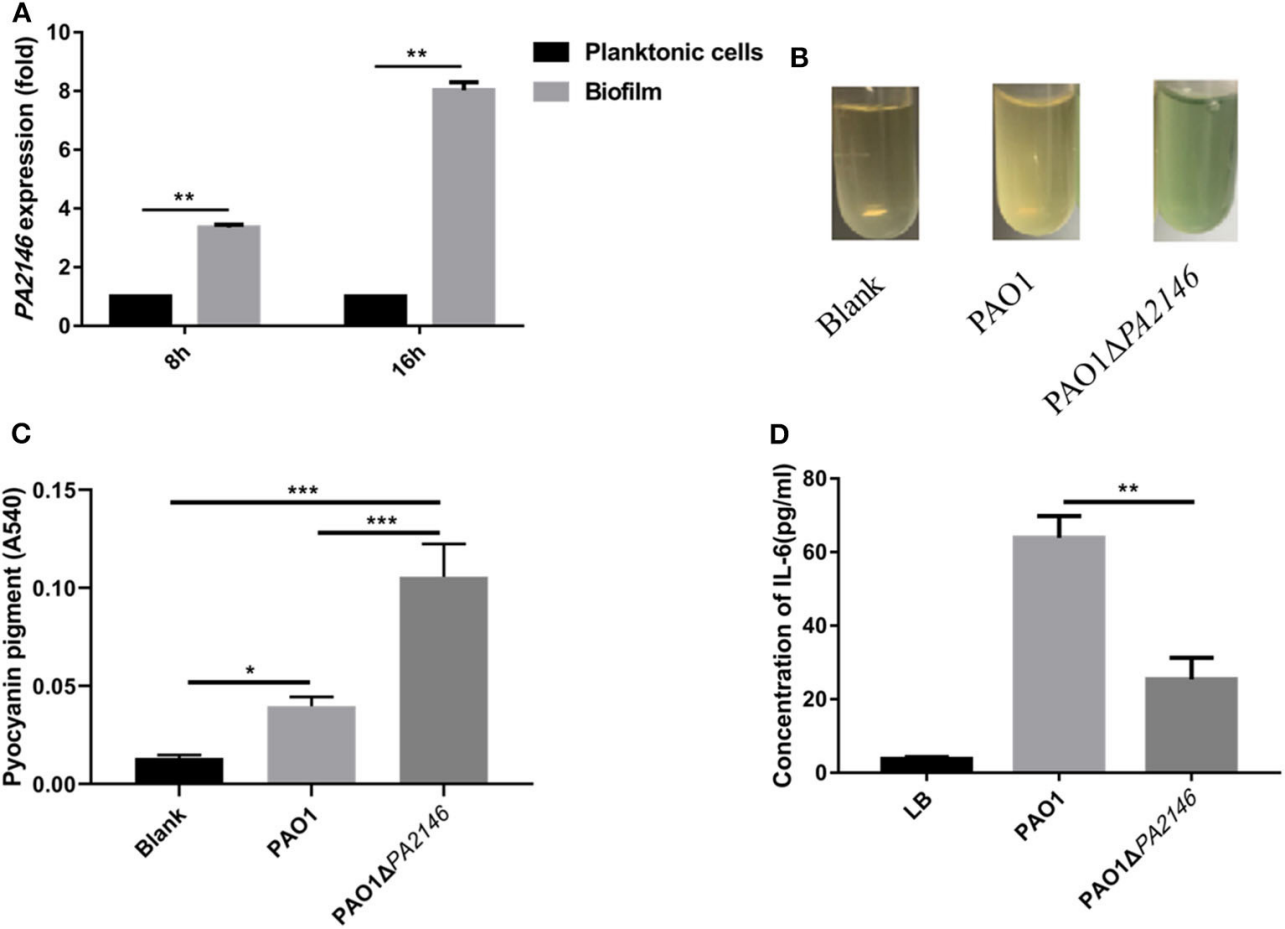
**(A)** *PA2146* expression in *P. aeruginosa* biofilms and planktonic cells after 8 and 16 h of incubation. **(B)** Representative images of pyocyanin production in PAO1 and PAO1Δ*PA2146* strains after overnight incubation. **(C)** Quantification of pyocyanin pigment by chloroform and hydrogen extraction, the absorbance at 540 nm was detected. **(D)** IL-6 production by neutrophils after incubation with PAO1 and PAO1Δ*PA2146* supernatant for 12 h. *P < 0.5; **P < 0.01; and ***P < 0.001. **(A,B,D)** Based on a study first published by our group in *Journal of Pathogen Biology*, 2020, 15:141–145 (Chinese version).

*Pseudomonas aeruginosa* secretes a variety of virulence factors and forms biofilms, which are mainly regulated by the QS system (Wang et al., 2020), and *PA2146* was found to be a gene related to the QS system. In our previous study, we found that *PA2146* was more highly expressed in biofilms than in its planktonic counterpart after 8 and 16 h of incubation (Figure 1A). This indicates that *PA2146* may be a crucial gene in the biofilm formation of *P. aeruginosa*. In order to explore its physiological function in *P. aeruginosa*, we constructed the *PA2146* knockout strain PAO1Δ*PA2146*. Our results showed that the production of pyocyanin pigment was significantly higher in PAO1Δ*PA2146* than in the PAO1 wild type strain (Figures 1B,C), indicating that *PA2146* may interact with the QS system of *P*. aeruginosa. However, IL-6 production by neutrophils was significantly reduced after stimulation with PAO1Δ*PA2146* culture supernatant compared to the PAO1 strain (Figure 1D).

### *PA2146* Deletion Has No Impact on RAW264.7 Phagocytosis

**Figure 2:**
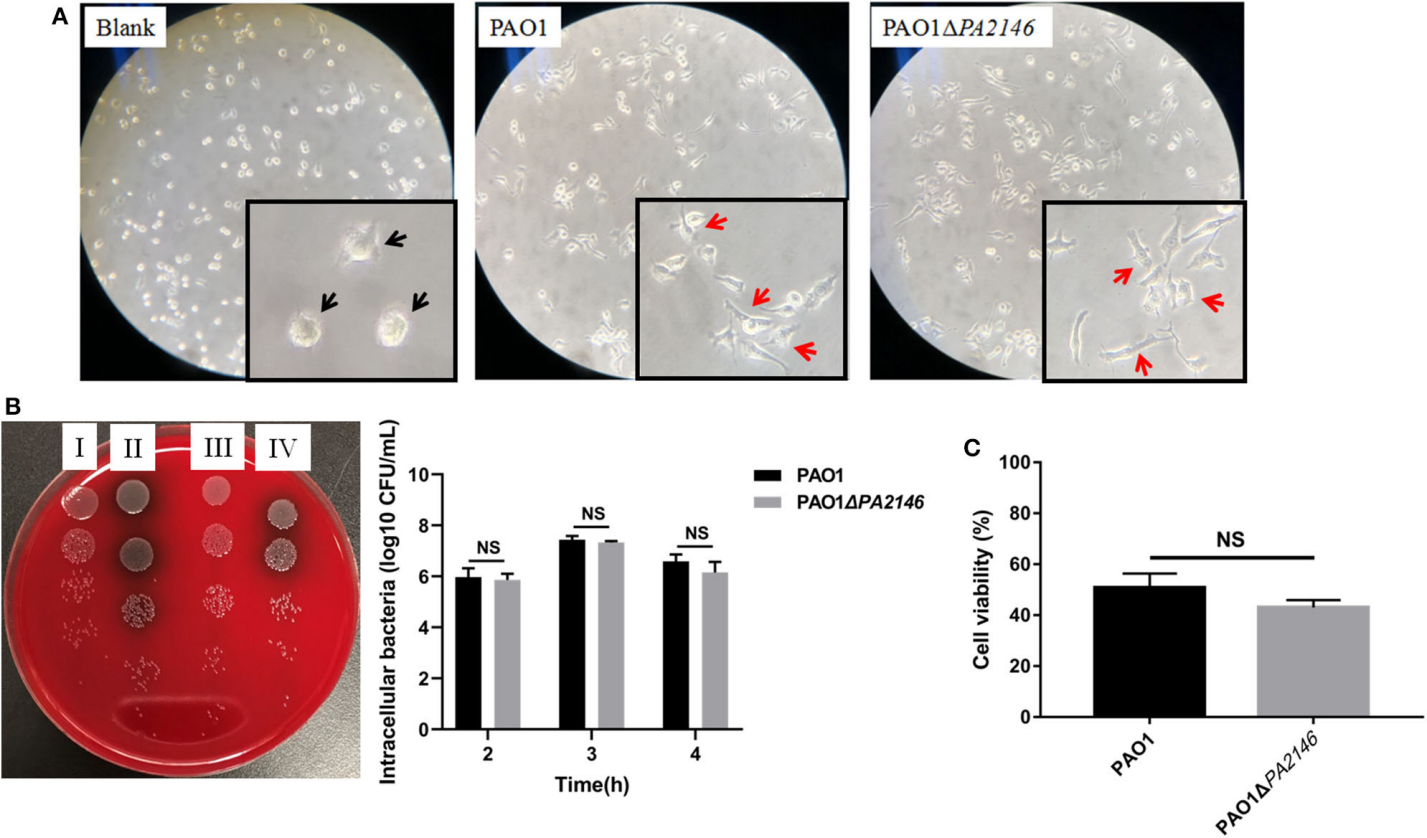
Effects of *PA2146* deletion on RAW264.7 phagocytosis. **(A)** Morphological alteration of RAW264.7 cells after stimulation with PAO1 and PAO1Δ*PA2146* at a MOI of 10 for 3 h. The black arrows indicate normal cells, and the red arrows indicate pathological cells with morphological changes like irregular cell shape pseudopods formation. **(B)** Phagocytic activity of RAW264.7 cells after stimulation with PAO1 and PAO1Δ*PA2146* at a MOI of 10 for 2, 3, and 4 h, respectively. Left panel: a representative image of 3 h treatment. (I) Intracellular and extracellular bacterial counts of PAO1; (II) intracellular and extracellular bacterial counts of PAO1Δ*PA2146*; (III) intracellular bacterial counts of PAO1; (IV) intracellular bacterial counts of PAO1Δ*PA2146*. Right panel: live bacterial cells counting. **(C)** Relative cell viability of RAW264.7 cells after treatment with PAO1 and PAO1Δ*PA2146* supernatant for 3 h. NS indicates no statistical significance.

Phagocytic cells play a central role in eliciting responses to acute *P. aeruginosa* infection. Phagocytosis can kill bacterial cells and present antigens to other immune cells (Pryjma et al., 1994). Here, we found that both PAO1 and PAO1Δ*PA2146* stimulation induced pathological and morphological changes in RAW264.7 cells, including intraphagocytic bacteria (Supplementary Figure 1), irregular cell shape, flattened and extended pseudopods while the PAO1Δ*PA2146*-treated group showed almost the same irregular cells as the PAO1-treated group (Figure 2A and Supplementary Figure 1). Though, the deletion of *PA2146* showed moderate tendency of inhibition activity against RAW264.7 phagocytosis-mediated killing (Figure 2B, Left panel), there was no statistical significance between wild type of PAO1 and PAO1Δ*PA2146* at 2 to 4 h treatment (Figure 2B, Right panel). Similarly, although the PAO1 or PAO1ΔPA2146 supernatant treatment significantly reduced the cell viability of RAW264.7 cells, there was still no statistical significance between the two groups (Figure 2C).

### Inhibition Cytokine Production in RAW264.7 and THP-1 Cells by *PA2146* Deletion

**Figure 3:**
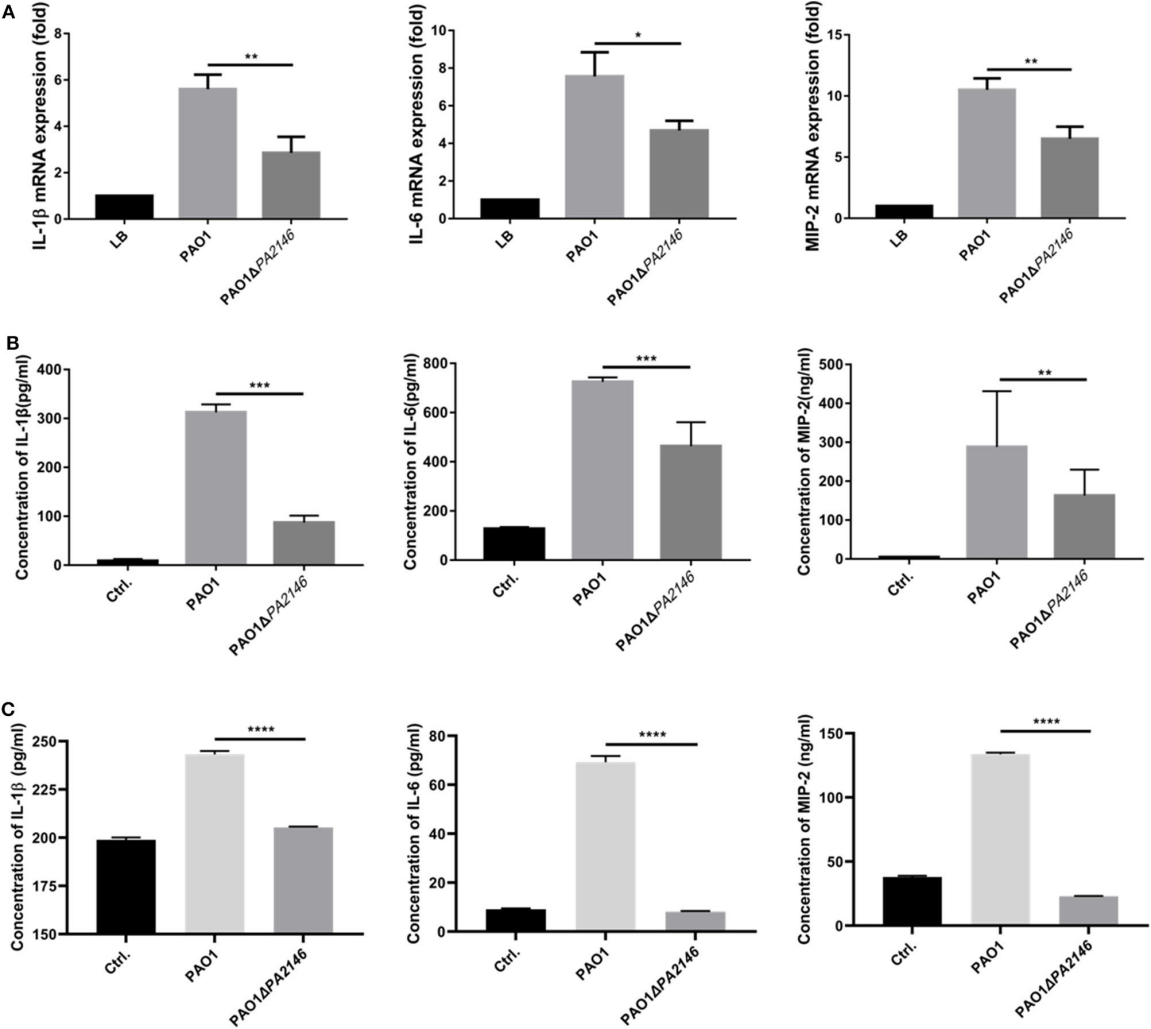
Effects of *PA2146* deletion on cytokine expression and production in RAW264.7 and THP-1 cells. The mRNA expression and production of the cytokines, IL-1β, IL-6, and MIP-2, by RAW264.7 were detected by qRT-PCR **(A)** and ELISA **(B)**, respectively. **(C)** The cytokines production by THP-1 were also detected by ELISA. The cells were stimulated with PAO1 and PAO1Δ*PA2146* supernatant for 3 h at the MOI of 10, cells stimulated with LB broth were set as control group. *P < 0.05; **P < 0.01; ***P < 0.001; and ****P < 0.0001.

**Figure 4:**
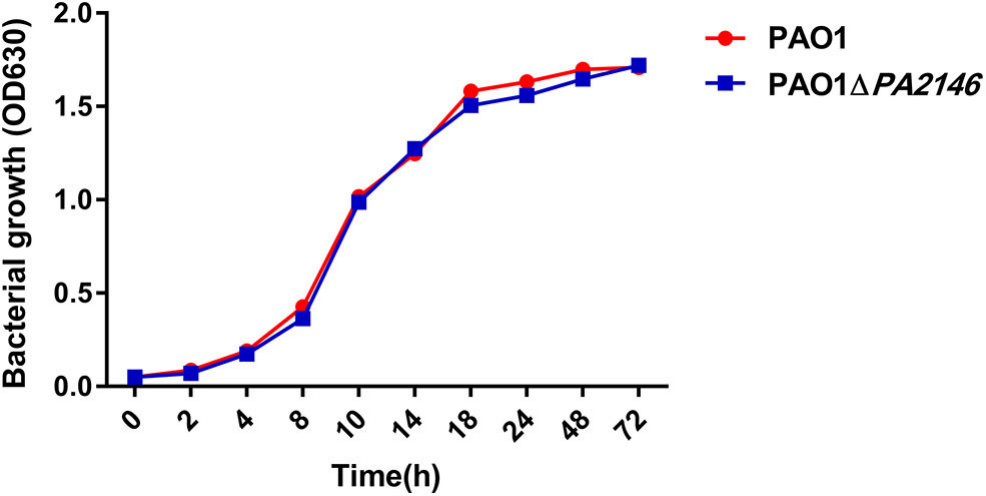
The growth curves of PAO1 and PAO1Δ*PA2146*. Strains were diluted with LB broth to ~ 1 × 10^5^ CFU/mL and incubated at 37°C 180 rpm.

Although *PA2146* deletion does not affect the phagocytic function of phagocyte cells, it significantly changes the production of cytokines in RAW264.7 and THP-1. The results showed that the cytokine levels increased significantly after treatment with *P. aeruginosa* supernatant. However, after deletion of *PA2146*, the mRNA (Figure 3A) and protein (Figure 3B) expression levels of the cytokines, IL-1β, IL-6, and MIP-2, in RAW264.7 cells were significantly reduced in *PAO1*Δ*PA2146-*stimulated group than those in the PAO1-stimulated group. Similarly, the production of cytokines in THP-1 cells were also significantly decreased after stimulated with *PA2146* deleted PAO1 than its wild type (Figure 3C). This indicates that the production of *PA2146*-related virulence factors may inhibit cytokine production and alter cell phenotype through cell receptors without affecting cell viability. In addition, no difference in cell growth was observed between the PAO1 and PAO1Δ*PA2146* groups (Figure 4), suggesting that the difference in cytokine secretion was not due to the different growth rates of *P. aeruginosa*.

### *PA2146* Deletion Promotes *P. aeruginosa* Pathogenicity *in vivo*

**Figure 5:**
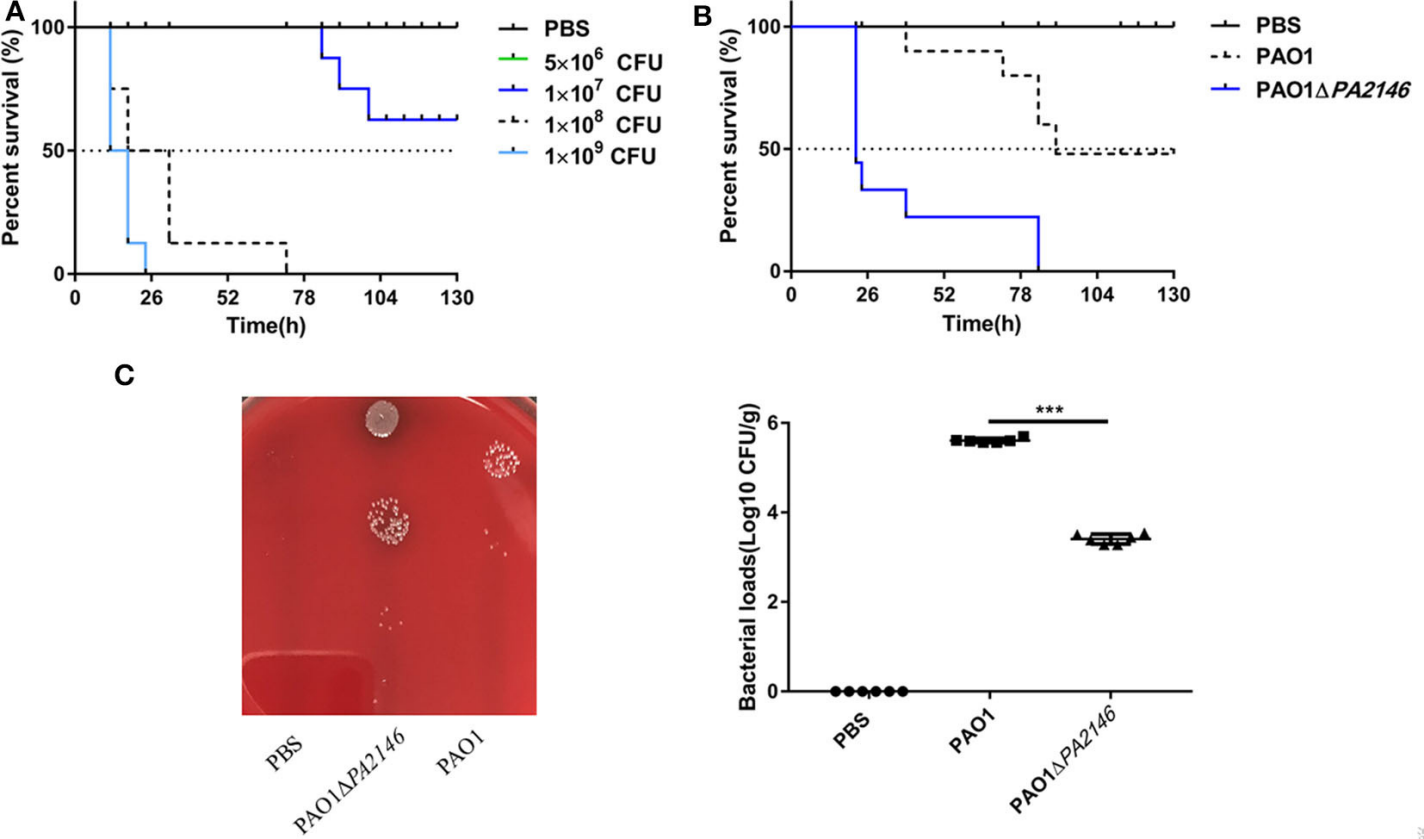
Effects of *PA2146* deletion on animal survival and bacterial colonization *in vivo*. **(A)** Survival rate of mice after treatment with PAO1 at different concentrations of inoculation ranging from 5 × 10^6^ to 1 × 10^9^ CFU per mouse (*n* = 8 mice/group). **(B)** Survival rate after treatment with PAO1 and PAO1Δ*PA2146* strains (*n* = 8 mice/group). **(C)** Bacterial loads in the mouse lungs after treatment with 1 × PBS, PAO1 and PAO1Δ*PA2146* for 12 h. ***P < 0.001.

We analyzed the pathogenicity and inflammation-inducing ability of PAO1 and PAO1Δ*PA2146* strains by constructing an acute lung infection model. To determine the optimal bacterial inoculum load, BALB/c mice were inoculated with 60 μL of PAO1 by tracheotomy operation, which containing a series of bacterial loads ranging from 5 × 10^6^ to 1 × 10^9^ CFU. As shown in Figure 5A, treatment with 5 × 10^6^ CFU of PAO1 did not cause any mice death; however, the survival rate of BALB/c mice was significantly reduced after treatment with 1 × 10^8^ and 1 × 10^9^ CFU PAO1 for 48 h. Thus, 1 × 10^7^ CFU/mice was chosen as the optimal inoculum load, considering that PAO1Δ*PA2146* treatment may cause a higher mortality than PAO1 treatment. As expected, the survival rate of PAO1ΔPA214 group was significantly lower than that of PAO1 group (Figure 5B). Similarly, the bacterial loads in the lungs of the PAO1Δ*PA2146* group were significantly higher than those in the PAO1 group (Figure 5C), suggesting that *PA2146* deletion may significantly increase bacterial colonization and/or virulence factor production, with higher lethality in the wild-type group.

### *PA2146* Deletion Increases Inflammation but Inhibits Cytokine Production *in vivo*

**Figure 6:**
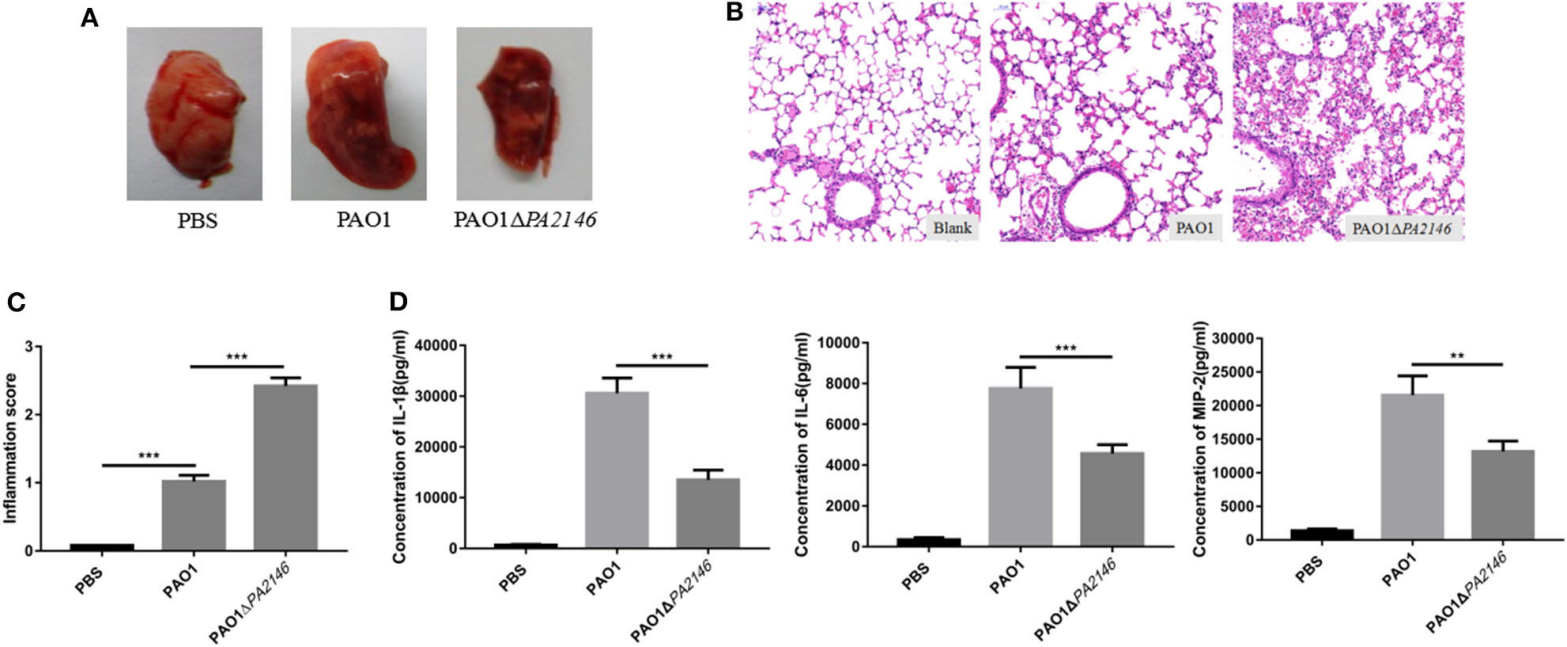
Evaluation of inflammation in mouse lungs after 20 h of infection with PAO1 and PAO1Δ*PA2146* at the inoculation of 5 × 10^6^ CFU/mouse. **(A)** Morphology of the lungs after treatment with 1 × PBS, PAO1 and PAO1Δ*PA2146* strains. **(B)** H&E staining results and **(C)** inflammation scores of the lung tissues after infection with 1 × PBS, PAO1 and PAO1Δ*PA2146*. **(D)** Production of the cytokines, IL-1β, IL-6, and MIP-2, in the lung homogenates, as detected by ELISA. **P < 0.01 and ***P < 0.001.

We constructed an *in vivo* model of acute *P. aeruginosa* associated pulmonary infection and found that *PA2146* deletion caused more severe hemorrhage in mice with the lung infection than in the wild-type group (Figure 6A). H&E staining and inflammation scores calculating revealed that the infiltration of erythrocytes and inflammatory cells in PAO1Δ*PA2146* group was significantly higher than that in PAO1 group (Figures 6B,C). However, lower levels of cytokines (IL-1β, IL-6, and MIP-2) were observed in the lungs of PAO1Δ*PA2146* group than in the lungs of PAO1 group (Figure 6D). This indicates that loss of *PA2146* leads to increased recruitment of inflammatory cells and inhibits the release of inflammatory factors.

## Discussion

*Pseudomonas aeruginosa* can cause a variety of infections, which often involves the synergy of multiple virulence genes and regulatory factors (Gellatly and Hancock, 2013). Although the DNA sequence of the *P. aeruginosa* genome has been recorded, the biological functions of a large number of genes remain unknown. In the present study, we characterized the virulence function of gene *PA2146* and its role in macrophage immune response and pathogenicity in an acute murine pneumonia infection model.

The virulence factors of *P. aeruginosa*, including pyocyanin, are mainly regulated by the QS system (Lee and Zhang, 2015). Deletion of QS-related genes (such as *lasR, rhlR*, or *pqsE*) could significantly inhibit the pyocyanin production (O'Loughlin et al., 2013). However, in our present study, the loss of PA2146 has been shown to significantly enhance PAO1 pyocyanin production; thus, *PA2146* could be a member of negative regulating genes of QS system. In addition, Attila et al. (2008) identified *PA2146* as a PAO1 virulence gene to poplar tree using transcriptome analysis and isogenic knockout mutants in a rhizosphere infection model. Dötsch et al. (2012) also reported that *PA2146* is more highly expressed in 24 and 48 h biofilms than its planktonic counterpart. Since the virulence factors and biofilm formation process of *P. aeruginosa* are both regulated by the QS system, we further hypothesized that *PA2146* may be a QS-related gene, which mainly down-regulating the production of pyocyanin and maybe other virulence factors.

At first, we speculated that the increased production of pyocyanin and other QS-related virulence factors may enhance the cytotoxicity against macrophage and further inhibit the phagocytosis-mediated bacterial killing effect. Although, both PAO1 and its *PA2146* knockout mutants showed significant toxicity to macrophage compared with control group, there is virtually no statistical difference for cytotoxicity or bacterial killing ability to macrophage between the two groups (Figure 2). Increased expression of QS-related signal molecular or virulence factors like N-(3-oxododecanoyl)-L-homoserine lactone may cause cytotoxicity to macrophage (Santajit et al., 2020) and thereby inhibiting their phagocytic function. However, pyocyanin is known to inhibit the inflammatory response of immune cells to mediate immune escape (Jayaseelan et al., 2014). Therefore, *PA2146* gene knockout promotes the production of pyocyanin, mediates the immune escape of macrophages, and neutralizes the toxicity of QS related signal molecules or other virulence factors.

Here, pa2146 deletion may downregulates macrophage immune response, possibly by increasing the production of pyocyanin. Pyocyanin is a type of pigment secreted by *P. aeruginosa* in the mid-log and stable growth phases and regulated by QS system (Lau et al., 2004). As mentioned above, pyocyanin can inhibit the inflammatory response of immune cells, reduce the flow rate of bronchial mucus and cilia, thereby promoting the colonization of Pseudomonas aeruginosa in the respiratory epithelium. Moreover, pyocyanin was also shown to enhance *P. aeruginosa* biofilm formation to escape immune cell attack (Jayaseelan et al., 2014). Healy et al. (2002) reported that high doses of pyocyanin could inhibit specific T and B cell immune responses, thereby enhancing the development of chronic infection. In this study, our results showed that *PA2146* deletion significantly increased pyocyanin production by *P. aeruginosa*. We speculate that this may be due to its immune escape function in inhibiting the secretion of cytokines by phagocytes. However, although cytokine secretion was inhibited in the lungs by *PA214*6 deletion, more severe lung damage and higher immune cell infiltration were observed in the PAO1Δ*PA2146* group than in the PAO1 group. Similarly, Marreiro de Sales-Neto et al. (2019) showed that treatment with pyocyanin can reduce the production of cytokines in macrophages but does not affect the migration of leukocytes to the site of inflammation. These paradoxical results could be attributed to the fact that besides increasing pyocyanin production, *PA2146* gene deletion may also significantly increase the production of other virulence factors, such as elastase, alkaline protease, phosphatase C, and endotoxins, which not only increases the colonization of bacteria and infiltration of inflammatory cells but it can also cause “shock” of immune cells and strongly inhibit their function after infection. In addition, overproduction of virulence factors may cause direct damage to respiratory epithelial cells and the lung tissue, leading to severe hemorrhage and oedema.

Although the deletion of *PA2146* had no effect on phagocytosis, the cytokines secretion of phagocyte was significantly inhibited, and the organs of mice were significantly deteriorated. However, the potential mechanism of PA2146 on immune cells is still unknown. Moreover, the regulatory mechanism of *PA2146* in *P. aeruginosa* also remains unclear and needs to be investigated in our future study. Based on our current findings, which highlight the function of *PA2146* in immune response in animal models, PA2146 could serve as a potential anti-virulent target for *P. aeruginosa*-related infections.

## Data Availability Statement

All datasets generated for this study are included in the article/Supplementary Material.

## Ethics Statement

This animal study was reviewed and approved by the Ethics Committee of the Third Xiangya Hospital of Central South University.

## Author Contributions

YL and PS performed the experiments, wrote the main manuscript text, and analyzed the data. YW, ZL, LC, and LZ helped to perform the experiments. YW, PS, and ZL conceived the experiments. LC, LZ, and ZH helped with the discussion of results. All authors reviewed the manuscript.

## Conflict of Interest

The authors declare that the research was conducted in the absence of any commercial or financial relationships that could be construed as a potential conflict of interest.

## Supplementary Material

Supplementary Figure 1 Representative images of RAW264.7 phagocytosis against PAO1 **(A)** and PAO1Δ*PA2146* **(B)**, respectively. RAW264.7 cells were infected with bacterial cells at a MOI of 10 for 3 h, and stained with safranin. The black arrows indicate the intraphagocytic bacteria. Scale bar: 50 μm.
