# Supplementary figures and images for: PA2146 Gene Knockout Is Associated With Pseudomonas aeruginosa Pathogenicity in Macrophage and Host Immune Response
Source: Front Cell Infect Microbiol. 2020 Oct 7;10:559803. doi: 10.3389/fcimb.2020.559803 (PMC7579411; doi:10.3389/fcimb.2020.559803)

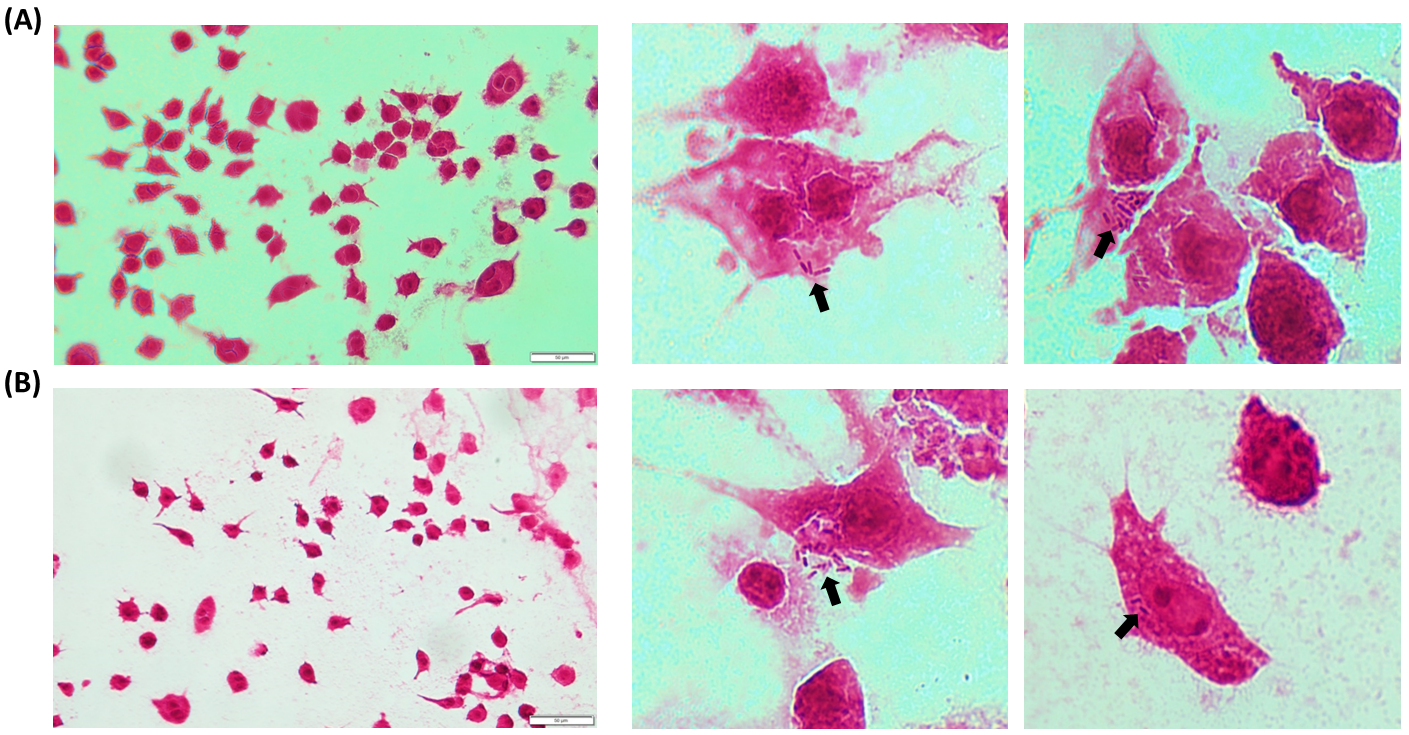

Supplement: Supplementary Figure 1 — Representative images of RAW264.7 phagocytosis against PAO1 (A) and PAO1ΔPA2146 (B), respectively. RAW264.7 cells were infected with bacterial cells at a MOI of 10 for 3 h, and stained with safranin. The black arrows indicate the intraphagocytic bacteria. Scale bar: 50 μm. [file Image_1.tif]
